# Peri-Ictal and Ictal Cognitive Dysfunction in Epilepsy

**DOI:** 10.3233/BEN-2011-0320

**Published:** 2011-03-29

**Authors:** Lavanya Vijayaraghavan, Subbulakshmy Natarajan, Ennapadam Srinivas Krishnamoorthy

**Affiliations:** The Institute of Neurological SciencesVoluntary Health Services Medical CentreRajiv Gandhi SalaiTaramaniChennaiTamil NaduIndia

**Keywords:** Cognitive dysfunction, ictal, peri-ictal, seizures, epilepsy

## Abstract

Disturbances in cognitive function, particularly memory, are a common complaint of patients with epilepsy. Factors contributing to cognitive dysfunction are the type of epilepsy, type and frequency of seizures, anti-epileptic drugs and the location of underlying brain lesions. Whilst a great deal of attention has been paid to permanent cognitive impairment, the nature and underlying mechanisms of ictal and peri-ictal cognitive changes are poorly understood. In-depth investigation of seizure related cognitive dysfunction is of great clinical relevance, as these changes are potentially reversible and treatable, thus reducing the cumulative effect of frequent seizures Greater knowledge of peri-ictal and ictal cognitive dysfunction would improve seizure prediction, localization of seizure focus and assessment of treatment effectiveness, greatly reducing distress and disability. This paper will review current understanding of peri-ictal and ictal cognitive dysfunction and discuss future directions for research.

## 1. Introduction

Disturbances in cognitive function, particularly memory, are common complaints of patients with epilepsy and result in considerable distress and disability [2,13]. Cognitive dysfunction in epilepsy may be either permanent and irreversible, resulting from congenital brain damage or temporary and potentially treatable, resulting from on-going seizure activity (state-dependent cognitive dysfunction). It is extremely important to distinguish clearly between permanent and reversible changes in order to identify the most appropriate form of treatment. In 1994, it was noted that the concept of state-dependent, or seizure-related cognitive dysfunction was less widely acknowledged compared to permanent cognitive impairment related to congenital brain damage in epilepsy [8]. Furthermore, repeated seizures may have a cumulative effect on the brain, resulting in decline [2]. Therefore, the focus of this review will be on cognitive changes that occur in the pre-ictal., i.e., 24 hour period preceding seizure onset, ictal, i.e., during a seizure and post-ictal., i.e., 24 hour period following seizure offset.

A two-way relationship between seizures and cognition has been described. Seizures may cause disruption of cognitive functions, yet some cognitive processes may themselves precipitate seizures, i.e., reflex epilepsies. Furthermore, engaging in some types of cognitive tasks may even inhibit seizure generation [35]. Cognitive precipitants of seizures have been discussed in detail in recent reviews [9,26] and will not be addressed here. A clear definition of the various terms and concepts addressed in this review will be followed by a discussion of current understanding of peri-ictal and ictal cognitive dysfunction.

### 1.1. Distinguishing seizures from epilepsy

Traditionally, both seizures and epileptic syndromes have been classified as ‘focal’ and ‘generalized’, with further classification of syndromes according to the underlying cause, i.e., idiopathic, symptomatic and cryptogenic [12]. The most recent Commission on Clas-

ISSN 0953-4180/11/$27.50 © 2011 – IOS Press and the authors. All rights reserved sification of the International League Against Epilepsy (ILAE) has recommended that only seizures will be referred to as focal and generalized and that epileptic syndromes will be classified according to the etiological cause, i.e., genetic, structural/metabolic and unknown [7].

Focal and generalized seizures, differing as they do in phenomenology, are likely to have different cognitive consequences. Ictally, focal seizures may affect specific cognitive functions due to involvement of specific neural substrates of those functions, whilst generalized seizures may have a global effect due to loss of consciousness. Furthermore, mechanisms underlying the transition from inter-ictal to ictal states may be different in focal and generalized seizures, thus influencing pre-ictal and post-ictal presentation of cognitive impairment.

### *1.2. Investigation of cognitive dysfunction in epilepsy*

Epilepsy researchers have divided cognition into 'trait' vs. 'state' functions. Trait functions require accumulation of knowledge over long periods, are assessed by measures of educational achievement and vocabulary, are less likely to be associated with focal brain lesions and are more likely to show gradual and chronic loss [2,42]. 'State' functions are more transient, are recruited according to the current situation and are more likely to be affected in an acute, limited and reversible manner following focal and generalized seizures [42]. These 'state' functions include types of attentional processes such as focused, divided and sustained attention, learning and memory [6,44], executive functions [21, 20,22] and language [6,14,40].

Focused attention is the selective processing of relevant information whilst screening out distraction. For example, in the Stroop Colour-Word Naming Task, subjects are asked to name the incongruent print colour of colour names; for e.g., the word RED is printed in green ink; subjects have to suppress their response to the green ink and read out the colour word red. Divided attention is the ability to simultaneously perform two tasks; for e.g., rapidly alternating between following numerical and alphabetic series in the Trail Making Test (B). Sustained attention is tested by assessing changes in reaction time and accuracy during a lengthy but simple task.

Verbal and visuo-spatial memories are tested by presenting words or pictures respectively during the seizure; encoding is tested by recall of items verbally or by drawing after the patient is oriented [44].

'Executive function' is a complex concept and considered synonymous with frontal lobe function. However, most tasks of 'frontal lobe/executive function' lack specificity; impaired performance can be seen in patients with brain lesions that exclude the frontal lobe [3]. The common element of executive function tasks is flexible selection and initiation of responses that are appropriate for changing behavioural contexts and suppression of inappropriate responses, as required by the Wisconsin Card Sort Task and its variants. Language includes comprehension and production components, which are tested by the execution of verbal commands and naming objects respectively [6].

### *1.3. Current understanding and knowledge of peri-ictal and ictal cognitive dysfunction*

Important reasons for evaluating cognitive function in epilepsy are subjective complaints of cognitive impairment, particularly memory; pre-surgical evaluation aimed at localizing the seizure focus and assessing the effect of anti-epileptic drugs on cognitive function. Searching the online data base Pub Med with the keywords peri-ictal, pre-ictal, ictal, post-ictal in conjunction with cognitive, neuropsychology, language, attention, memory and executive function retrieved only a few studies where the ictal state was confirmed using EEG when cognitive testing was carried out [2,6,14, 31,32]. Retrospective studies were included to identify the types of cognitive dysfunction reported in the pre-ictal period, which may then help to plan more systematic studies that test specific hypotheses. Anecdotal case reports, which may or may not include evaluation with standardized neuropsychological tests were also retrieved, were interpreted cautiously. Factors that may explain the paucity of systematic investigation of the acute effects of seizures are unpredictability and short duration of seizures, except for frequently occurring absence seizures and non-convulsive status epilepticus. Furthermore, systematic cognitive testing during the peri-ictal period requires hospital admission and video-EEG monitoring, which are expensive and may not be available on a routine basis.

#### *1.3.1. Pre-ictal cognitive deficits*

A retrospective study involving semi-structured interviews of 100 patients with epilepsy and their informants revealed that prodromal symptoms were reported by 35% of patients [41]. Cognitive and behavioural symptoms were the two most common prodromal events. Cognitive symptoms included 'bradipsychia', increased latency in verbal and motor responses, clumsiness, short-term memory and attention disturbances, whilst irritability was the most commonly reported affective/behavioural symptom. Prodromal symptoms (PS) were related to seizure frequency, but not type, etiology or age at onset. PS appeared in a variable period of time (generally several hours) before seizure onset and lasted until seizure onset, suggesting that the pre-ictal period may be regarded as a developing process that culminates in a seizure. Despite the weaknesses of this study, such as its retrospective nature and lack of EEG confirmation of the pre-ictal state, the findings indicate that further research in this hitherto neglected area is necessary.

*1.3.2. Ictal cognitive deficits*

The impact of seizure activity on cognitive function depends on the type of seizure, i.e., focal vs. generalized and on the presence of focal structural lesions. Focal seizures are more likely to disrupt specific domains of cognitive function; for e.g., selective impairment of memory in patients with focal seizures with temporal lobe pathology [10,11,44] and impairment of language in seizures with a left inferior frontal locus [14]. Generalized seizures, such as absence seizures that are of short duration and high frequency impair attentional processes. Cognitively, a patient with absence seizures may become unresponsive or perseverate in the task being performed [2].

However, in a large study of primary school children (aged 7–12 years), Aldenkamp and colleagues did not find an effect of seizure type (focal vs. generalized) on either trait or state measures of cognitive function [2]. Instead, there was an effect of presence of seizures during testing on such as memory, processing speed and reaction time. Furthermore, increased number of seizures had an effect on reaction time tasks, whilst poor performance on verbal memory tasks was associated with duration of seizures. Seizure-related variables did not influence trait cognitive functions; instead, the children with partial epilepsy of cryptogenic or symptomatic etiology showed delayed educational achievement compared to children with idiopathic generalized epilepsy and healthy children.

Understanding the effects of seizures on cognitive function may be complicated by the fact that loss of consciousness is not an all-or-none phenomenon; some cognitive functions may be affected whilst others may be intact [6]. Lux and colleagues [32] examined the effect of seizures on four constituents of consciousness in 116 patients with intractable seizures who were undergoing pre-surgical assessment. These included orientation (ability to turn towards the examiner on sensory stimulation), expressive speech (words and sentences without dysphasic errors), responsive speech (appropriate verbal or non-verbal responses to verbal requests) and memory (details of the seizure or ictal examination post-ictally). Video-EEG recordings confirmed that seizures were occurring during cognitive testing; seizures were focal and did not generalize. Patients were divided into four groups based on the putative focus of seizures: frontal, left temporal, right temporal and bi-temporal.

Bi-temporal foci were associated with impairment of all four constituents, left temporal foci with impairment in expressive and receptive speech but not orientation, frontal foci with impairment in orientation and expressive speech but not receptive speech and memory. In striking contrast, orientation, receptive and expressive speech and memory were preserved in subjects with seizures arising from the right temporal lobe.

Lux and colleagues [32] concluded that normal function of both temporal lobes seemed to be important for fully preserved consciousness, without impairment in speech, behaviour or memory. However, the individual contributions or relative importance of the left vs. right temporal lobes are unclear. It may be possible that this study did not include tests that were more sensitive to and specific of right temporal dysfunction.

Transient epileptic amnesia (TEA) is an ictal cognitive phenomenon that has received considerable attention recently [10,11,33,34,43,44]. In 1996, Vuilleumier and colleagues [44] reported a classic case study of middle-aged woman who suffered from multiple episodes of retrograde as well as anterograde amnesia on waking, often preceded by an epigastric aura. The ictal nature of these episodes was confirmed by EEG recordings of 3.5–4 Hz spike-wave activity, which was abolished by intravenous clonazepam.

Patients with TEA are generally middle aged or elderly and experience brief, recurrent episodes of anterograde and/or retrograde amnesia that often occur on awakening. Generally, other cognitive functions seem unimpaired. Episodes of TEA may be either the sole manifestation of seizures, or may follow focal seizures with impairment of consciousness and automatisms (complex partial seizures). TEA may be confused with transient global amnesia, which is a vascular phenomenon or with so-called hysterical attacks; however the presence of epileptiform abnormalities on electroencephalography, the concurrent onset of other manifestations of seizures such as lip-smacking or olfactory hallucinations and a clear-cut response to anticonvulsant therapy can definitively distinguish TEA from other conditions [11].

Memory impairment in TEA can involve patchy loss of memory for events in the past, usually autobiographical, accompanied by difficulty in recognition of famous faces and names [44]. TEA is also characterized by accelerated forgetting, where subjects can encode and recall new information for short periods of time but seem to forget faster compared to healthy subjects for verbal but not for non-verbal items [33,34]. EEG recordings of patients with TEA have demonstrated epileptiform activity localized to either left or right temporal lobes, or bitemporally [44]. Often, neuroimaging does not reveal any lesions, but when they are present, they are more likely to be found in the medial temporal lobes [11].

It has been proposed that TEA is an ictal phenomenon with continuation into the post-ictal phase. Ictal activity in the medial temporal structures may impair encoding of new memories (anterograde amnesia) and retrieval of past memories (retrograde amnesia). Hypoxic damage to the medial temporal lobes associated with cardiovascular disease may be an underlying pathogenic mechanism.

Aphasia is another ictal cognitive phenomenon, associated with seizures with left temporal lobe foci. Patients suffering from refractory focal seizures with automatisms and impaired consciousness (complex partial seizures) with EEG confirmed foci in left temporal lobes demonstrated slower reading time during a seizure than patients with right-sided foci. Furthermore, post-ictal paraphasias were observed only in subjects with left-sided foci [40].

Patients with a frontal focus of seizures (frontal lobe epilepsy, FLE) show a cognitive profile that is quite different from patients with temporal lobe seizures. Helmstaedter and colleagues [20] identified four 'core' processes that form the basis of most tasks of frontal lobe function: psychomotor speed/attention, motor coordination, working memory and response inhibition. Impairment in motor skills and response inhibition were the two deficits that significantly differentiated patients with frontal lobe epilepsy (FLE) from patients with temporal lobe epilepsy (TLE) in the inter-ictal period.

Frontal lobe seizures are associated with expressive speech impairments accompanied by impairment of orientation reflexes, motor automatisms. However, receptive speech and consciousness are preserved to some extent and patients can remember details of testing during the episode [31,32]. In frontal non-convulsive status epilepticus, patients appear slow, dysphoric and morose. Loss of self-initiated behaviour, perseverations, intrusion of irrelevant information, apraxia, difficulty in shifting performance from one task to another [20]. Comparing frontal seizures and non-convulsive frontal status, Helmstaedter [21] hypothesized that the motor and other manifestations of frontal seizures are related to direct excitation of frontal areas. In contrast, hyper-inhibition during non-convulsive status results in impaired executive control of behaviour.

Seizures and cognitive deficits may also arise in situations of metabolic stress. A case report by Dinner and colleagues [14] describes de novo sudden onset of severe aphasia and confusion in a 60 year-old diabetic. Aphasia was receptive and expressive, with impaired comprehension and failure to respond to verbal commands, and persisted in a milder form in the inter-ictal period. EEG recorded during an aphasic episode confirmed seizure activity that migrated from the inferior frontal to posterior temporo-occiptal regions. The inter-ictal aphasia probably was a post-ictal manifestation of metabolic exhaustion of neurons of the speech area rather than due to an underlying structural lesion. Phenytoin and insulin therapy resulted in improvement of aphasia and discharge of a neurologically normal patient 6 weeks after admission.

#### *1.3.3. Post-ictal cognitive deficits*

Post-ictal cognitive deficits may be a continuation of the ictal cognitive impairment into the post-ictal state. They may also be a separate phenomenon, related to wide-spread inhibition of neuronal activity and metabolic exhaustion of neurons. The duration of the immediate post-ictal state can be tracked by serum prolactin and cortisol levels that peak 10–20 minutes after a seizure and return to baseline within 60–90 minutes [36]. The delayed post-ictal state may manifest after a symptom free period ranging from several hours to days [25]. Cognitive and behavioural changes during the delayed post-ictal state may be mistaken for inter-ictal phenomena.

Post-ictal aphasia, manifesting as language delay and paraphasias has been reported following seizures that originate in the temporal lobe of the dominant hemisphere and spread to Broca's and Wernicke's areas [1] as well as after seizures originating in the non-dominant temporal lobe with contralateral spread [18].

Unilateral spatial neglect (USN), or a failure to report, respond, or orient to stimuli that are presented contralaterally is another cognitive deficit that is present in the post-ictal but not in the interictal state. Pripilko and colleagues [39], administered a line-bisection task to 33 patients with refractory focal seizures (15 left and 18 right), with foci primarily in the temporal lobes; a few patients had parietal and frontal foci. In the line-bisection task, subjects are asked to bisect a series of horizontal lines of different length drawn at different locations on a sheet of paper. The subjective centre of the line identified by subjects with right hemisphere lesions tends to deviate to the right. There was no difference in task performance between patients and healthy controls in the inter-ictal phase. In the immediate post-ictal state, patients with right-sided foci (temporal or frontal) tended to erroneously bisect the line to the right side of the centre of the line, but only for lines drawn on the left side of the sheet. Subjects with left-sided foci did not show an effect of focus location or line position. The authors strongly recommend that symptoms of neglect should be actively looked for, as impaired performance in the line-bisection task was noticed only post-ictally and not in the inter-ictal state.

Post-ictal cognitive deficits, when focal, contribute to fine-tuning the localization of seizure focus identified via EEG. The lateralization of verbal and visual memory functions to the left and right temporal lobes respectively is an issue of great relevance. Post-ictal testing has shown that verbal memory is affected following left temporal seizures and that visual memory is affected following right temporal seizures [31]. Furthermore, secondary generalization was associated with greater severity of the deficit. Also, patients with frontal foci did not show impairment in either visual or verbal memory.

Post-ictal cognitive testing may be of greater value than inter-ictal testing, particularly during pre-surgical evaluation [4,37,39]. Andrewes and colleagues [4] reported that inter-ictal testing of verbal and visual memory failed to distinguish between patients with right vs. left-sided temporal foci undergoing evaluation for epilepsy surgery. When re-tested within 1 hour of an EEG confirmed seizure, patients with right temporal foci showed the expected pattern impaired visual but not verbal memory and vice versa. The side of seizure focus predicted by task performance was confirmed post-operatively.

There are few reports of post-ictal deficits in frontal lobe function. This state of affairs may be due to the relatively lower prevalence of seizures with frontal lobe origin [21]. Underlying lesions, when present, are heterogeneous in nature and location. Also, ictal activity originating in the frontal lobes tends to spread very rapidly to other brain regions, due to the large number of excitatory projections from the frontal lobes to the rest of the brain. Furthermore, patients with frontal lobe seizures re-orient sooner than patients with temporal lobe seizures and rarely show cognitive deficits such as reaction time in the post-ictal period [31,32].

### *1.4. Neurobiological mechanisms underlying the peri-ictal and ictal states*

A two-stage model of seizure generation is generally accepted, where there is an abrupt transition from the non-seizing inter-ictal state to the ictal state. Seizure initiation begins with a loss of inhibitory control of epileptogenic zone and spreads as neighbouring neurons become increasingly excitable. Synaptic excitation and inhibition that results in depolarization of neurons followed by strong hyper-polarization has been proposed as a mechanism underlying seizure termination [38].

However, there is increasing clinical and neurophysiological evidence that the change may be more gradual, involving a pre-ictal transition state, which then leads to a seizure [5,17,23]. The transition from post-ictal to inter-ictal may be gradual as well; EEG abnormalities such as focal or diffuse polymorphic delta activity, attenuation or activation or focal spikes have been demonstrated upto several hours after a seizure [24]. Recent investigation of cortical excitability using Transcranial Magnetic Stimulation (TMS), has demonstrated significantly increased excitability of the motor cortex in the 24 hours preceding seizure onset and decreased excitability in the 24 hours following seizure offset in drug-naïve, newly diagnosed patients with epilepsy [5]. Patients with generalized seizures had bilateral changes in excitability, whilst those with focal seizures had ipsilateral changes in excitability.

Apart from clinical seizure states, these processes of hyperpolarization and inhibition with associated cognitive and behavioral changes may also be existent subclinically, as response to provocative factors in the environment, or to the introduction/ alteration of AED therapy. Indeed, the mechanisms that underlie the electrophysiological phenomenon of Forced Normalization of Landolt and the corresponding clinical phenomenon of Tellenbach's alternative psychoses have been well described [27,28]. The interface between forced normalization phenomena and cognitive functioning has not been studied in detail, but may putatively involve processes similar to those described peri-ictally herein. Their clinical importance lies in the relative frequency of sub-clinical phenomena such as these in the person with epilepsy.

The neurophysiological changes underlying the peri-ictal and ictal state have also been measured non-invasively. Measurement of regional and global cerebral blood flow with PET and SPECT in patients with refractory focal seizures have demonstrated a distinct pattern of blood flow change in the inter-ictal, ictal and peri-ictal states [15,16,29,37]. In patients with focal seizures, the inter-ictal state is characterized by regional hypoperfusion in the putative locus. Regional hypoperfusion changes to either increase or decrease in the pre-ictal state, hyperperfusion in the ictal state and returns to hypoperfusion in the post-ictal state. In patients with suspected medial temporal lobe foci, the ictal state is characterized by uniform and global ipsilateral temporal lobe hyperperfusion. Within a few minutes of cessation of seizures, the picture of uniform global hyperperfusion switches suddenly to a combination of ipsilateral hippocampal and medial temporal hyperperfusion and lateral temporal hypoperfusion.

Pre-ictal imaging with BOLD fMRI has demonstrated a very complex pattern of BOLD signal intensity changes in patients with refractory focal seizures [Federico]. BOLD signal intensity may increase focally around the presumed seizure focus or may show widespread ipsilateral increase or decrease, accompanied by contralateral increase in BOLD signal. The relationship of BOLD signal increase and decrease to neuronal excitation and inhibition is highly complex and not well understood [30]. However, the authors speculate that signal decreases near the seizure focus could indicate suppression of pre-ictal neuronal activation by active inhibitory processes from other brain regions. The attempt to suppress activation by distal brain regions could be reflected as increase in contralateral BOLD signal.

## 2. Conclusions

There is increasing evidence to support the concept that transition from inter-ictal to ictal states may be gradual and consist of pre and post ictal periods that have distinct clinical, electrochemical, metabolic, behavioural and cognitive profiles. These profiles may be modified by the type of seizure (focal vs. generalized), type of epilepsy, location of seizure focus and/or underlying lesion and concurrent treatment with antiepileptic drugs. Cognitive impairment in the peri-ictal period may be due to loss of information input due to global loss of consciousness or due to impaired processing of information due to disruption of neural networks that mediate said processing. Seizures are likely to disrupt so-called 'state' cognitive functions, which are transient, recruited according to the current situation and are more likely to be affected in an acute, limited and reversible manner. 'State' functions that have been affected by seizures include language, learning & memory, attention, decision-making, visuo-spatial function and response inhibition. Cognitive impairment may be restricted to individual domains in the case of focal seizures. Memory is disproportionately affected by focal seizures with temporal lobe foci, particularly medial. Furthermore, the laterality of seizure focus may have specific effects on cognitive processes, i.e., verbal memory impairment with left temporal foci and visual memory impairment with right temporal foci. Aphasia may be total, receptive or expressive and has been reported in the ictal and post-ictal states, with seizure foci in the inferior frontal and lateral temporal cortices. Right parietal-temporal seizures have been associated with unilateral neglect, which is not detectable during the inter-ictal phase.

## 3. Future directions

Despite reviews emphasizing the lack of knowledge regarding the cognitive consequences of peri-ictal and ictal states [9,19], there is a striking paucity of publications regarding this neglected aspect of epilepsy. There is an urgent need for world-wide, prospective studies that examine the relationship between seizures and cognitive processing in people with epilepsy. Apart from routine neuropsychological testing, cognitive tasks aimed at testing specific hypotheses together with continuous EEG recordings are necessary to identify and confirm peri-ictal and ictal states and to validate the results.

Baseline brain function during various peri-ictal and ictal states can also be studied with PET, SPECT or BOLD-fMRI and compared to those acquired when the subject is performing a cognitive task, revealing the response of the brain during a seizure to cognitive challenges.

Greater understanding of the clinical, electrochemical, metabolic, behavioural and cognitive aspects of the peri-ictal and ictal periods could increase the efficacy of treatment. Pre-surgical cognitive evaluation in the ictal and peri-ictal period could contribute to localizing seizure foci, confirming the laterality of the focus and identify cognitive changes that are absent in the interictal state, thus contributing to increased success of epilepsy surgery. Furthermore a quantitative measure of cognitive deficit associated particularly with seizures may help in post-surgical evaluation.

Identifying the cognitive consequences of seizures and the underlying pathological mechanism would contribute greatly to the development of new anti-epileptic drugs (AED). The cognitive side-effects of AEDs could be distinguished from the cognitive consequences of seizures, thereby improving the selection of appropriate AEDs. Again, quantitative measurements of cognitive deficits in the peri-ictal and ictal periods may be a valuable indicator in trials of AED efficacy. Furthermore, better characterization of seizure-related cognitive dysfunction could suggest new avenues for rehabilitative therapies.

In conclusion, it is hoped that in the future, peri-ictal and ictal states are no longer “neglected entity (ies) in the management of epilepsy”, as noted by Fisher and Schacter [19].

## Acknowledgements

The authors would like to acknowledge the financial support from the T.S. Srinivasan Institute of Neurological Sciences during the time this paper was written.
